# Surfactants: physicochemical interactions with biological macromolecules

**DOI:** 10.1007/s10529-020-03054-1

**Published:** 2021-02-03

**Authors:** M. Aguirre-Ramírez, H. Silva-Jiménez, I. M. Banat, M. A. Díaz De Rienzo

**Affiliations:** 1 Departamento de Ciencias Químico Biológicas, Instituto de Ciencias Biomédicas, Universidad Autónoma de Ciudad Juárez, Ciudad Juárez, Chihuahua, Mexico; 2 Área de Oceanografía Química, Instituto de Investigaciones Oceanológicas, Universidad Autónoma de Baja California, Ensenada, Baja California Mexico; 3 School of Biomedical Sciences, University of Ulster, Coleraine, BT52 1SA Northern Ireland, UK; 4 School of Pharmacy and Biomolecular Sciences, Liverpool John Moores University, James Parsons Building 10.05C, Byrom Street, Liverpool, L3 3AF UK

**Keywords:** Surfactants, Macromolecules, Biological systems, Molecular interactions

## Abstract

Macromolecules are essential cellular components in biological systems responsible for performing a large number of functions that are necessary for growth and perseverance of living organisms. Proteins, lipids and carbohydrates are three major classes of biological macromolecules. To predict the structure, function, and behaviour of any cluster of macromolecules, it is necessary to understand the interaction between them and other components through basic principles of chemistry and physics. An important number of macromolecules are present in mixtures with surfactants, where a combination of hydrophobic and electrostatic interactions is responsible for the specific properties of any solution. It has been demonstrated that surfactants can help the formation of helices in some proteins thereby promoting protein structure formation. On the other hand, there is extensive research towards the use of surfactants to solubilize drugs and pharmaceuticals; therefore, it is evident that the interaction between surfactants with macromolecules is important for many applications which includes environmental processes and the pharmaceutical industry. In this review, we describe the properties of different types of surfactants that are relevant for their physicochemical interactions with biological macromolecules, from macromolecules–surfactant complexes to hydrophobic and electrostatic interactions.

## Introduction

Surfactants are amphiphilic molecules capable of reducing the surface tension between two immiscible phases (Otzen 2017). These molecules are either chemically produced (synthetic surfactants) or based on biological materials (biosurfactants). The reduction of surface tension is due to their amphiphilic properties, as their molecules consist of both hydrophilic and hydrophobic moieties (Li and Lee 2019). The hydrophilic part contains heteroatoms such as oxygen, sulphur, nitrogen and phosphorous, which appear in functional groups such as alcohol, thiol, ether, ester, acid, sulphate, sulfonate, phosphate, amine, amide, etc., while the hydrophobic part is typically a paraffin, cycloparaffin or aromatic hydrocarbon, which may contain halogens. Due to their dual affinity, amphiphilic molecules are not stable either in polar or in organic solvents. To meet both types of affinities, the hydrophilic moiety must be surrounded by a polar solvent, while the hydrophobic moiety must be in contact with an organic solvent. Such conditions exist only between two immiscible phases. The boundary between a condensed phase and a gaseous phase is referred to as a surface, and the boundary between two condensed phases such as two liquids or a liquid and a solid, is referred to as an interphase. Many properties of surfactants depend on this strong affinity for surfaces or interphases (Khan et al. 2015).

There are important properties that characterise each particular system. Surface tension is defined as the work required to increase the area of a surface isothermally and reversibly by unit amount (Ebnesajjad 2014). Surface tension (γ) is expressed as surface energy per unit area and alternatively as a force per unit length. If we consider two identical phases the surface tension (${\gamma }_{1})$ can be expressed by Eq. 1:

1 $${\gamma }_{1}= \frac{1}{2}{w}_{11}$$

where *W*_11_ represents the work of adhesion between the two identical phases, which is defined as the reversible thermodynamic work required to separate the interface from the equilibrium state of the two phases to a separation distance of infinity.

On the other hand, the interfacial tension between two different phases (1 and 2) can be given by Eq. 2:

2 $${\gamma }_{12}= {\gamma }_{1}+ {\gamma }_{2 } - {w}_{12}$$

These characteristics are determinant in terms of the properties of the systems, such as the existence and persistence of emulsions or foams, where surfactants are responsible for the changes (reduction) in surface tension. Surfactants allow the mixing of hydrophilic molecules with hydrophobic ones, through the formation of structures called micelles which allow the association of both types of molecules in a single phase. This compatibility between molecules that do not have a natural affinity is also known as co-solubilisation (Poša et al. 2019) and can be used to establish different applications.

Surfactants are used in a wide range of industrial applications (Banat and Thavasi 2018). In agriculture, for example, phytosanitary agents are applied in the form of aerosol (surfactant) which, sometimes, contains a dispersed organic phase (emulsifier) to dissolve herbicides and insecticides (Marquez et al. 2018). While in food products, they contribute to the conditioning of creams, suspensions, emulsions, soluble or dispersible powders (Kralova and Sjöblom 2009). In mining processes, they play an important role in the flotation and leaching of metals like iron, zinc, uranium (Asselin and Ingram 2014; Diaz et al. 2015); as well as in the textile industry to improve the performance of different operations and to provide particular properties to the finished products (Pacifico and Giers 1995; Proffitt and Patterson 1988). In the oil industry, they have been used to help to solve problems caused by drilling operations to the conditioning of the finished products; in fact, extracted crude oil reaches the surface in the form of a water-in-oil emulsion, which makes it essential to remove or separate the water content (Marquez et al. 2019).

Chemical surfactants are derived from non-biodegradable components, and in some cases can cause serious problems to the environment, such as: (1) the formation of foams which inhibit or paralyze natural (or artificial) purification processes, concentrate impurities and can spread bacteria or viruses; (2) the increase of phosphate content in basins, from polyphosphates that are used in combination with surfactants (Santos et al. 2016).

**Fig. 1:**
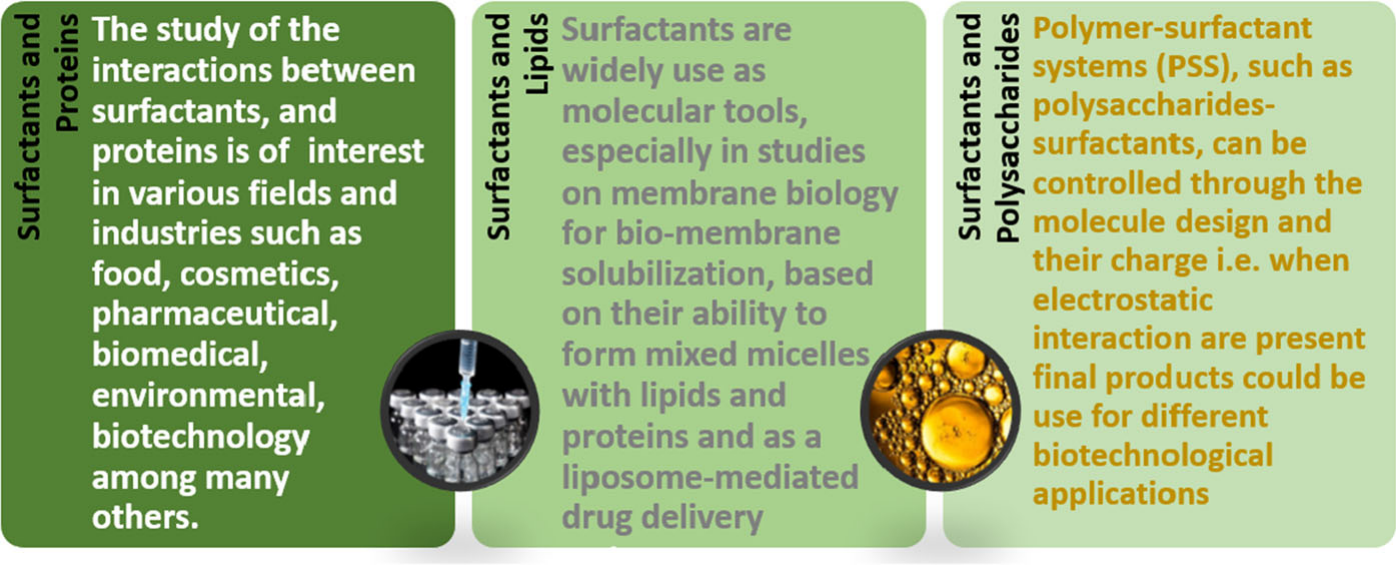
Illustrative summary of the main types of interactions between (bio)surfactants and macromolecules

Given the problems caused by synthetic surfactants, different studies have been carried out over the past years, seeking to find alternative products compatible with the environment and have demonstrated the feasibility of producing these compounds from microorganisms (Akbari et al. 2018). Most microbial biosurfactants are typically biodegradable, biocompatible and have stable activities under extreme environmental conditions (Naughton et al. 2019). Hence the interest to study their production from fungi and bacteria, among which the genera *Bacillus* and *Pseudomonas* stand out. Many of these biosurfactants produced by *Pseudomonas aeruginosa* have been characterized and studied as agents capable of removing hydrophobic compounds from soil (Geetha et al. 2018), antimicrobials and biofilm disruptors (Elshikh et al. 2017; Diaz De Rienzo et al. 2016; Ceresa et al. 2020). Although the physicochemical properties of (bio) surfactants have been well documented through the years (Mankowich 1953; Behrens 1964; Van Os et al. 1993; Patino et al. 2007; Morais et al. 2017), their interaction with biological components has had less focus. This review therefore focuses on the properties of surfactants that are relevant for their physico-chemical interactions with biological systems (Fig. 1), and when possible compare them with their biological counterparts.

## Surfactant–protein interactions

The study of the interactions between surfactants, both synthetic and microbial (biosurfactants), with proteins is of great interest in various biotechnology fields and industries such as food, cosmetics, pharmaceutical, biomedical, and environmental (Lee et al. 2011; Otzen 2011; Tucker et al. 2014; Malik 2015). In the biomedical industry, protein–surfactant systems are used for the production of hydrogels (Afinjuomo et al. 2019; Castelli et al. 2008). The hydrogels form the base of fibrous proteins such as fibroin, which are used for tissue regeneration and drug delivery (Park et al. 2014; Dubey et al. 2018; Ohadi et al. 2020).

There are three main forces that drive the protein–surfactant interaction: (1) electrostatic, (2) hydrophobic and (3) Van der Waals (Mackie and Wilde 2005; Li and Lee 2019). The dominant interaction is determined by the nature of both molecules and their concentration (Mehan et al. 2015; Li and Lee 2019). These molecular interactions have an influence on the native structure of proteins promoting or preventing denaturation, aggregation and loss of enzymatic activity among other factors (Mehan et al. 2015). Surfactants of biological origin have an advantage over synthetic surfactants in terms of their ability to prevent denaturation of proteins and a reduction in their aggregation (Otzen 2011, 2017).

The protein–surfactant systems mainly studied are those that contain globular proteins such as bovine serum albumin (BSA), α-lactoglobulin and β-glucosidase. In contrast, very few studies have been performed exploring the fibrous protein–surfactant systems. Type I collagen, silk fibroin, and keratin are fibrous proteins that have been studied in combination with ionic and non-ionic surfactants (Maldonado et al. 1991; Mandal and Kund 2008; Kezwon et al. 2016; Kezwoń and Wojciechowski 2016; Pan et al. 2016; Park et al. 2014; Dubey et al. 2018). A few studies suggest that the molecular interactions presented by fibrous proteins (collagen, fibroin, keratin) in combination with ionic and non-ionic surfactants are similar to the globular protein–surfactant systems (Lee et al. 2011; Khan et al. 2015, Kezwon et al. 2016; Kezwoń and Wojciechowski 2016; Pan et al. 2016).

Type I collagen interacts with Sodium Dodecyl Sulphate (SDS), Cetyl Trimethyl Ammonium Bromide (CTAB), and Triton X-100 through hydrophobic and electrostatic molecular interactions. The predominance of a particular molecular interaction depends on the type of surfactant, i.e. surfactants could produce changes in collagen secondary structure (Maldonado et al. 1991; Kezwon et al. 2016; Kezwoń and Wojciechowski 2016).

The main physical parameters that have an effect on the surfactant–protein interactions are: (a) the surfactant concentration; (b) the chemical nature of surfactant (ionic or non-ionic surfactants); and (c) the secondary structure of the protein (α-helix and β-sheets) (Díaz et al. 2003; Malik 2015).

### Surfactant concentration

The effect on stabilization or destabilization mediated by a surfactant is dependent on the concentration of the surfactant (Mehan et al. 2015). In that sense, many surfactants (biological and synthetic ones), usually promote protein stabilization at concentrations far below Critical Micelle Concentration (CMC), while at concentrations higher than the CMC there is an opposite effect, they promote denaturation, aggregation, as well as loss of biological function of proteins (Díaz et al. 2003; Otzen 2011; Malik 2015). In general, the binding of the surfactant to the protein is carried out in three phases. In the binding phase (phase I), individual surfactant molecules bind to the protein without causing any structural change, and electrostatic interactions dominate over hydrophobic ones. In the cooperative phase (phase II), the increase in the surfactant concentration reaches a sub-CMC levels, triggering the formation of the hydrophobic clusters that start to bind to the hydrophobic regions of proteins leading to their denaturation and changes in the secondary structure. In this phase, hydrophobic interactions dominate over electrostatic; in addition, the unfolding process increases linearly (Otzen 2011; Malik 2015). Finally, the saturation phase (phase III) is where the protein binding sites are already saturated. In this phase, there are free surfactant molecules that interact with the protein-bound micelles and no longer cause further changes (Malik 2015).

### Chemical nature of surfactants

Surfactants can be divided into two groups according to their chemical composition: ionic and non-ionic. The ionic surfactants, according to their charge, can be anionic or cationic (Otzen 2011; Khan et al. 2015). The hydrophilic group of the surfactant affects the stability of the protein because it can tightly bind to the protein causing its denaturation and contributes to the solubilization of the membrane proteins (Mehan et al. 2015). Anionic surfactants are typically protein-denaturing agents (Khan et al. 2015). Among the anionic surfactants, SDS is well known for having strong electrostatic interactions with proteins (Deep and Ahluwalia 2001; Otzen et al. 2009; Hansted et al. 2011; Otzen 2011). These interactions are generated between the positively charged amino acids present in the primary structure of the protein along with the interactions of the hydrocarbon chains of the surfactant, and the aliphatic regions of the amino acids arginine (Arg) and lysine (Lys) (Otzen et al. 2009).

**Fig. 2:**
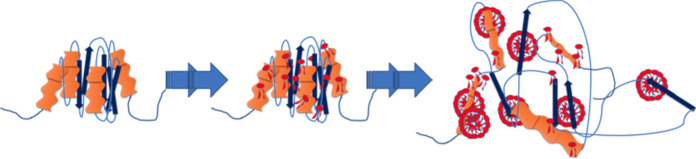
Representative scheme of the denaturation effect promoted by SDS over α-lactalbumin. SDS monomers bind to the protein starting the denaturation process; at a high concentration of SDS monomers, the secondary structure of the protein is lost

Such properties have been used in some protein separation and/or solubilisation techniques. The interaction between SDS and several globular proteins has been previously reported, i.e. the denaturing effect of SDS on α-lactalbumin occurs in different stages depending on the concentration of the surfactant. In the early stages, SDS monomers bind to the protein to form groups up to a critical concentration that results in the start of the denaturation process (Fig. 2). The binding of more monomers results in the loss of the secondary structure of the protein (Otzen et al. 2009). In the case of β-lactoglobulin, SDS has an opposite effect to the one observed with α-lactalbumin, since this amphiphilic molecule reduces the aggregation of the protein at concentrations well below its CMC (Hansted et al. 2011).

Compared to anionic surfactants, cationic surfactants have a milder protein destabilization effect (Khan et al. 2015). These ionic surfactants interact with amino acids whose side chains are usually negatively charged like aspartate (Asp) and glutamate (Glu) (Otzen 2011). For example, Khan et al. (2019) reported that the interactions between CTAB and Hen egg white lysozyme are very hydrophobic and weakly electrostatic, which do not cause a change in the secondary structure of the protein but do cause a negative effect on the tertiary structure.

In the case of non-ionic surfactants (i.e., dodecyl maltoside, polysorbates), they commonly minimize or prevent protein aggregation (Lee et al. 2011; Otzen 2011). According to various studies, the molecular interactions between proteins and non-ionic surfactants are very weak and the union of the biomolecule with the non-ionic surfactant is driven by hydrophobic interactions, which results in a tendency to solubilize proteins. These surfactants are used in the food industry and have biomedical applications in drug formulations (Lee et al. 2011; Campos et al. 2013; Tucker et al. 2014). Non-ionic surfactants usually have ethoxylate groups that interact with the hydrophobic moieties of proteins, exposing the hydrophilic groups present in both molecules, which results in the increase of the hydrophilicity of the non-ionic surfactant–protein complex, thereby reducing the aggregation of proteins (Rudolph and Jones 2002; Ruiz-Peña et al. 2010; Lee et al. 2011; Tucker et al. 2014). The chemical structure of this type of surfactant plays an important role in promoting or preventing protein denaturation, even if the structural differences are minor. Tween type surfactants (ethoxylated polysorbates) vary in the length of the fatty acid hydrocarbon chain and interact differently with BSA, as seen in the number of surfactant molecules that are able to bind to the protein as well as the type of binding (Ruiz-Peña et al. 2010).

Another type of surfactants known as dimeric or Gemini surfactants are constructed of two monomers of surfactants which are joined by a spacer close to the hydrophilic heads (Sinha et al. 2016). Despite their importance in several industrial fields, studies of Protein-Gemini surfactants interactions are limited, compared with those conducted with single chain surfactants (Sinha et al. 2016; Parray et al. 2018; Akram et al. 2019). Several studies have revealed that some interaction mechanisms of these new generation of surfactants with proteins are shared with their corresponding monomers differing in the effects that they induce in the biomolecule, ranging from having stronger molecular interactions than their monomeric counterpart to changes or stabilization in the secondary and tertiary structures of proteins (Sinha et al. 2016; Sonu et al. 2017; Akram et al. 2019). Comparative studies of the interaction of BSA with the cationic surfactant Dodecyl Trimethyl Ammonium Bromide (DTAB) and with three Gemini-surfactants of the bis(dimethyldodecylammonium bromide) family; butanediyl-1,4-bis(dimethyldodecylammonium bromide (12–4-12,2Br −), 2-butanol-1,4-bis(dimethyldodecylammonium bromide) (12–4(OH)-12,2Br −), 2,4-dibutanol-1,4-bis(dimethyldodecylammonium bromide) (12-4(OH)2-12,2Br −), showed that at lower concentrations of the surfactant the interaction in the surfactant–protein complex is managed by electrostatic forces and while the concentration of the surfactant increases.

The union of the protein with the surfactant is hydrophobic in nature, which is stronger with the Gemini-surfactant, causing greater denaturation of BSA compared to DTAB, which suggests that the spacer between the two monomers plays an important role (Sinha et al. 2016). Sonu et al. (2017) conducted a study on the effect of surfactant spacers [12-8-12, 2Br-], [12-4-12, 2Br-] and [12-4 (OH) -12, 2Br-] on the interaction with BSA and reported that the more hydrophobic the spacer is, the lower is the reduction in the number of α-helices and denaturing effects. Akram et al. (2019) on the other hand, analysed the interaction of the BSA model protein with three members of a family of Gemini Cm-E20-Cm surfactants and demonstrated that the binding of these dimeric surfactants with the protein is considerably strong, without causing a significant loss of α-helix (3–4%), keeping the secondary and tertiary structure of the BSA virtually intact. Other authors have reported that the effect caused by these Gemini-surfactants on the various model proteins may be subject to changes at different temperatures, pH concentrations, ionic strength, and surfactant concentrations, among others (Faustino et al. 2009).

### Secondary structure of proteins

In some cases, the secondary structure of a protein could have an effect on the ability of a surfactant to promote its aggregation or denaturation activities, without necessarily being a specific surfactant–protein interaction. Zaragoza et al. (2012) showed that when the trehalolipid biosurfactant produced by a *Rhodococcus *sp*.* is present at a concentration lower than CMC, proteins with a high content of α-helix in the secondary structure such as BSA and cytochrome *c* (Cyt-*c*) showed resistance to thermal unfolding and there was no alteration of the secondary structure. In addition, Isothermal Titration Calorimetry (ITC) investigations demonstrated that the interactions between trehalolipids and both proteins are not specific, suggesting the involvement of hydrophobic domains of proteins (Zaragoza et al. 2012). However, the biosurfactant mannosylerythritol lipid-A (MEL-A) has a different influence on the enzyme β-glucosidase. At CMC values, this biosurfactant promotes a secondary structure changes of β-glucosidase, causing a decrease in β-sheets content and an increase in α-helices, β-turn, and random coil. These structural changes cause β-glucosidase to acquire thermal stability by increasing its midpoint temperature (*T*_*m*_) and unfolding enthalpy (Fan et al. 2018).

The above can be explained in thermodynamic and structural terms. On the one hand, at CMC values, MEL-A forms micelles, thereby increasing hydrophobic interactions. Thermodynamic data obtained by ITC, support the hypothesis that weak hydrophobic interactions are responsible for the union of MEL-A and β-glucosidase. On the other hand, the stability gained by β-glucosidase at CMC values can be given by the enzyme’s secondary structural changes. The increase of α-helix content is a potential factor which promotes, (1) the exposure of hydrophobic regions to amino acid residues that interact hydrophobically, (2) hydrogen bond formation with fatty acid chains, and (3) hydroxyl groups of glycosidic residues (Otzen 2011; Fan et al. 2018).

Based on various analytical methods, Zhang and Li (2018) reported that surfactin, a biosurfactant of the lipopeptide type, induces changes in the conformations of the alkaline protease secreted by *Bacillus *sp., which results in weak hydrophobic interactions, hydrogen bonds and some electrostatic interactions. In addition, they found that the enzymatic activity of the alkaline protease may be affected positively or negatively at low or high concentrations of surfactin, respectively. In the first case, the low concentration of surfactin in the aqueous medium, allows the biosurfactant molecule to interact with the alkaline protease as a cofactor, thus causing an increase in enzymatic activity, while at high concentrations of surfactin, a decrease in enzymatic activity occurs. This is because the hydrophobicity of the alkaline protease is decreased by the high concentration of biosurfactant molecules present in the solution. Finally, the cases analysed in this review on the interactions between different surfactants with a model protein reveal that they are quite diverse, where the physicochemical characteristics of the interacting molecules play an essential role. Molecular interaction studies using various biophysical techniques, will allow us to understand the basis of interaction between surfactants and proteins.

## Surfactant–lipid interactions

The phase behaviour between surfactants–water and lipid–water is well documented (Chernik 2000; Koynova and Tenchov 2001; Ebnesajjad 2006), however the interaction between surfactants and lipids is not well reported with most studies have been carried out on temperature and enthalpy variables without a detailed description of the mechanisms involved (Koynova and Tenchov 2001). Surfactants are widely used as molecular tools, especially in studies of membrane biology for biomembrane solubilization, based on their ability to form mixed micelles with lipids and proteins (Koynova and Tenchov 2001) and as a liposome-mediated drug delivery system (Bnyan et al. 2018). Liposomes have been used as a model of biological membranes for a long time, due to their phospholipid structure. The structure of phospholipids has a hydrophilic head group and a hydrophobic tail group. When dispersed in an aqueous solution, the head is attracted by water, and the tail, including a long hydrocarbon chain, is repelled by water promoting the formation of vesicles (Stryer 1981; Dua et al. 2012; Gunay and Ozer 2018).

**Fig. 3:**
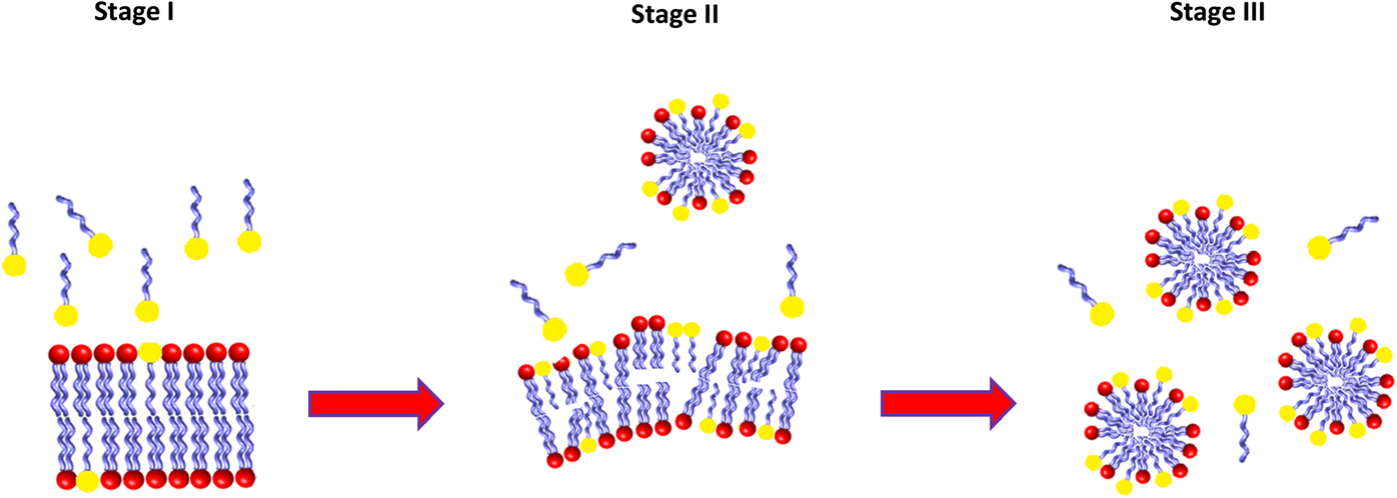
Surfactants–lipids interaction: the three-stage model. Stage I: Surfactant molecules approach a bilayer. Stage II: Combination of micelles and lipid/surfactant aggregates. Stage III: Mixed micelles formation

The interaction between lipids and surfactants is derived in a different numbers of model systems (Helenius and Simons 1975; Lichtenberg et al. 1983). All these models show a general scheme for the interaction between lipids and surfactants (which displays the transition from vesicles to mixed micelles) and is described as a three-stage model (Fig. 3). The first stage is where the surfactant partition between the lipid bilayers and the aqueous phase and start reaching a level where the bilayers break into micelles; the second phase is where there is a mix between micelles and bilayers in a co-existent state and the last phase is characterized by an increase of the surfactant concentration leading to a phase where all the bilayers are solubilized and only lipid-rich micelles are present (Lichtenberg et al. 2013; Pizzirusso et al. 2017).

There are different studies that show the three-stage model applied to biological membranes, including homogenous phospholipids systems (phosphatidylcholine and phosphatidylserine), Ca^2+^-ATPase membranes (Le Maire et al. 2000) and liposomes prepared from SR lipid (Langner and Hui 2000). The solubilisation of membranes generally occurs via the uptake of non-micellar surfactants monomers, which is why when a surfactant is added to solubilize a membrane preparation, if the surfactant concentration is below their CMC, then it is just the monomer fraction that interact with the biological membrane.

When it comes to the study of biosurfactants and membrane lipids interactions, few studies have been reported on molecular interactions (Ortiz et al. 2009; Aranda et al. 2007; Rodrigues et al. 2006; Malaspina et al. 2017). The effect of trehalose lipids on membrane phospholipids was reported by Ortiz et al. (2008) showing that the biosurfactants exhibit a dehydrating effect on the interfacial region of saturated phosphatidylethanolamines promoting the formation of unsaturated phosphatidylethanolamines. The same research group evaluated the effect of trehalose lipid produced by *Rhodococcus *sp. on the structural properties of dimyristoyl phosphatidylserine (DMPS) membranes. They have showed that the biosurfactant incorporates into the DMPS membranes and increases the fluidity of the phosphatidylserine acyl chains making changes in the environment of the polar head group and, as a consequence, decreases the interfacial tension of the membrane, thereby decreasing the motional freedom of the phospholipids (Ortiz et al. 2009).

One of the most studied biosurfactant in terms of their effect on the plasma membrane is the Iturin produced by *Bacillus subtilis*. Iturin is an effective antifungal compound and its mechanisms of action is related to the disruption of the biological membrane by the formation of small vesicles and their aggregation in yeast cells (Peypoux et al. 1994; Rodrigues et al. 2006). Iturin was shown to pass through the cell wall and disrupt the plasma membrane with the formation of small vesicles and the aggregation of intramembranous particles, interacting with the nuclear membrane and probably with membranes of other cytoplasmic organelles affecting the morphology and membrane structure of yeast cells (Thimon et al. 1995). Recently, the studies in molecular surfactant-like peptides and lipids has become more focused and significant due to their excellent properties, such as versatility, biocompatibility and medicinal properties (Cui et al. 2010; Hosseinkhani et al. 2013; Dehsorkhi et al. 2014; Du and Stenzel 2014; Malaspina et al. 2017; Doostmohammadi et al. 2019).

An important class of amphiphilic peptides called surfactant-like peptides (SLPs), present an intrinsic difference that can lead to different physical consequences namely composition and tail structure (Malaspina et al. 2017). Unlike conventional surfactants whose hydrophobic tails interact in all directions through hydrophobic interactions, the amphiphilic peptide tail contains not only hydrophobic groups but also hydrophilic sites (Colherinhas and Fileti 2014). This feature allows the SLPs to stabilize nanostructures in one direction through hydrophobic interactions and in the orthogonal direction by hydrogen bonds. These hydrogen bonds associated with hydrophobic interactions can stabilize at a high level, complex secondary structures such as helices and sheets. On the other hand, conventional lipids/surfactants with antimicrobials properties (Chen et al. 2010, 2012; Albada et al. 2012; Gaspar et al. 2013) are usually organized into micelles, vesicles, and nanotubes (Colherinhas and Fileti 2014; Malaspina et al. 2017). To understand the interaction between (bio)surfactants and lipids, it is necessary to be aware of the hydrodynamics of the molecules involved, their amphiphilic properties and how they play an important role when it comes to biological membranes. Nanoparticle models and the study of their properties could help us to understand the molecular basis of these interactions, which have remained unknown.

## Surfactant–polysaccharide interactions

Polysaccharides are monosaccharide (homo or hetero) built up biopolymers mainly produced by plants. Similar to surfactants, they could be classified based on their charge as non-ionic (o), cationic (+), and anionic (−) polymers (Kwak 1998). Polysaccharides and surfactant interactions are important to develop (a) emulsifiers; (b) flocculating agents; (c) stabilizing colloids; (d) or rheology controllers (Holmberg et al. 2002) in food, medicine and environmental applications.

Electrostatic, hydrophobic, dipole–dipole, and hydrogen bonding interactions along with the surfactant and polysaccharide characteristic are the main factors that affect the Polymer–Surfactant Systems (PSS) (Grządka et al. 2019). These interactions have been summarised in Table 1 (Bao et al. 2008). These authors studied the interactions of ionic surfactants (SDS and CTAB) with neutral, positively, and negatively charged polysaccharides [Methyl cellulose (MC), chitosan (CS) and κ-carrageenan (KC)], respectively.

**Table 1:** Interactions between methyl cellulose, chitosan and κ-carrageenan with ionic surfactants, SDS and CTAB (Bao et al. 2008)

Polysaccharide	Surfactant	Interaction		
		Hydrophobic	Electrostatic	Ion–dipole
Methyl cellulose	SDS	Strong		Weak
Chitosan		Medium	Strong	
κ-Carrageenan		Weak		
Methyl cellulose	CTAB	Strong		
Chitosan		Medium		
κ-Carrageenan			Strong	

According to the surfactant–polysaccharide combination, molecular interactions change. Therefore, strong hydrophobic and weak ion–dipole interactions are present in MC–SDS mixture. Moreover, in KC–SDS and CS–SDS, ionic interactions drive the binding process between surfactant and the polymer. Hydrophobic interactions are weak in KC–SDS, while in CS–SDS, polymer hydrophobic moieties interact with alkyl chains of the SDS. In the case of CTAB with MC and CS, only hydrophobic interactions are present, and strong electrostatic interactions allow binding between KC and CTAB.

In the case of non-ionic polysaccharide and anionic surfactant, as ethyl hydroxyethyl cellulose (EHEC) and SDS, respectively, the hydrophobic interaction between the polymer and SDS alkyl chain drives their association. Accordingly, SDS plays an important role because its presence or absence promotes the extent of EHEC–SDS cluster formation. For example, if SDS concentration is below the critical aggregation concentration (CAC), surface tension is reduced depending on SDS molecules, but when SDS concentration increases to at or above the CAC, EHEC adsorption is accelerated. In diluted solutions, the surface activity is strong (12 ppm of EHEC and 2 mM SDS), making this PSS a vehicle for drug delivery (Nahringbauer 1997).

Cationic surfactants such as DTAB, MTAB, and CTAB, interact with cellulose in the water interface. These cationic surfactants contain a different number of –CH_2_– groups in the alkyl chain, and their CMC varies with respect to alkyl chain length (CTAB > MTAB > DTAB). The chain length of this kind of cationic surfactants influences interaction behaviour with non-ionic polysaccharides such as cellulose. For example, CTAB–cellulose interaction is driven by hydrophobic interactions, while electrostatic interactions are very significant in interactions of MTAB and DTAB with cellulose, respectively.

In the case of interactions of polysaccharides such as dextrin and carboxymethylcellulose with cationic surfactant groups (DTAB, MTAB, CTAB), the behaviour is different, for the interaction between dextrin and CTAB, hydrophobicity drives the interaction while in the case of carboxymethylcellulose and CTAB, electrostatic interactions are very significant (Biswas and Chattoraj 1997a, b).

Another example of PSS with an anionic surfactant, sodium stearoyl lactylate (SSL), an anionic surfactant and κ-carrageenan (KC) polymer, both of which are important in the food industry, have a different behaviour in solutions and gels. SSL changes KC conformation due to electrostatic interactions and hindrance. In the gelation process (melting process), KC suffers a coil helix transition and, finally, helix–helix aggregation, modifying its melting enthalpy. SSL hinders KC helix–helix aggregation. But, at a high concentration of surfactant, SSL forms micelles (solutions and gels). The combination of hindrance and electrostatic repulsion promote conformational changes in KC, both in solutions and in gels. In solution, enthalpy decreases continuously at high SSL concentration range, while in gels, this parameter decreases at a specific SSL concentration (Ortiz-Tafoya et al. 2018).

In other cases, the interaction between a polysaccharide and surfactant depends on the alkyl chain length of the tensioactive molecule. Such is the case of CTAB homologues (CnTAB, where **n** is a carbon number in alkyl chain of surfactant) with cellulose nanocrystals, a negatively charged polysaccharide. When **n** = 12 and the surfactant concentration is high, electrostatic interactions are present and micelle formation occurs, while at **n** = 14–16 and a low surfactant concentration, micelles are formed, and flocculation process occurs at high CnTAB concentration (Table 2). These processes are driven in first instance by electrostatic interactions and by the hydrophobic interactions (Brinatti et al. 2016).

**Table 2:** Interaction of cetyl trimethyl ammonium bromide family (CnTAB) with cellulose nanocrystals (C = carbon number in alkyl chain of surfactant) (Brinatti et al. 2016)

	CnTAB interaction with cellulose nanocrystals	Micelle formation	Flocculation
C = 12	Electrostatic	High concentration	
C = 14	Electrostatic–hydrophobic	Low concentration	High concentration
C = 16	Electrostatic–hydrophobic	Low concentration	High concentration

### Polysaccharides–biosurfactants interactions

Some biosurfactants contain sugars in their structure such as glycolipids (e.g. rhamnolipids) and also interact with polysaccharides. In the food and pharmaceutical industries, pickering/stabilizing high internal phase emulsions (HIPEs) are very important as they are used in bioactive delivery. In these HIPEs three kinds of molecules interact: proteins–polysaccharides–biosurfactants. For example, zein–propylene glycol alginate mixed with rhamnolipids stabilize pickering emulsion in the oil-in-water interface. This emulsion system is formed by a 3D network of adsorbed and non-adsorbed particles, however the basis of molecular interactions amongst these molecules is unclear (Dai et al. 2019).

## Conclusions

Recent works in this area highlight the importance of the interactions between surfactants and macromolecules and their role in biological membranes. The structures that form in solution are driven by molecular interactions. There are three main forces that drive the protein–surfactant interactions: electrostatic, hydrophobic, and Van der Waals, while the dominant interaction is controlled by the characteristics of both molecules and their concentration. The interactions between lipids and surfactants are described as a three-stage model, starting with the surfactant partition between the lipid bilayers and the aqueous phase, reaching a level where the bilayers break into micelles and ending with the solubilization of bilayers. The characteristics of PSS, such as polysaccharides–surfactants, can be controlled through the molecule design and their charge i.e. presence of electrostatic interaction at opposite charge PPS where hydrophobic interactions are predominant in o/− and o/+ PPS, and where o/− interaction is stronger than o/+ PSS; these are some of the most powerful parameters to take into account in order to obtain the desired structures to be used for different applications. By modifying the interaction type and strength, as well as the concentrations of the molecules involved, the final product can be used for a wide variety of industrial formulations.

## List of symbols

*γ*_11_: Surface tension when two identical phases are considered
*γ*_1_: Surface tension of phase 1 i.e. liquid
*γ*_2_: Surface tension of phase 2 i.e. solid
*γ*_12_: Interfacial tension between two different phases
*w*_11_: Work of adhesion between two identical phases
*w*_12_: Work of adhesion between two different phases
